# Resting-State Connectivity Changes After Goal-Oriented Attentional Self-Regulation Training in Veterans With Mild Traumatic Brain Injury: Preliminary Findings from a Randomized Controlled Trial

**DOI:** 10.1089/neur.2022.0074

**Published:** 2023-06-29

**Authors:** Maria Kryza-Lacombe, Rachel Santiago, Anna Hwang, Sky Raptentsetsang, Brian A. Maruyama, Jerry Chen, Marissa Cassar, Gary Abrams, Tatjana Novakovic-Agopian, Pratik Mukherjee

**Affiliations:** ^1^ Mental Illness Research Education and Clinical Centers, Department of Veterans Affairs, San Francisco VA Health Care System, San Francisco, California, USA.; ^2^ Department of Veterans Affairs, San Francisco VA Health Care System, San Francisco, California, USA.; ^3^ University of California, San Francisco, San Francisco, California, USA.; ^4^ Palo Alto University, Palo Alto, California, USA.

**Keywords:** clinical trial, cognitive rehabilitation, fMRI, resting-state, traumatic brain injury

## Abstract

Mild traumatic brain injury (mTBI) can have lasting consequences on cognitive functioning and well-being. Goal-Oriented Attentional Self-Regulation (GOALS) training has been shown to improve attention and executive functioning, as well as emotional functioning, in veterans with chronic TBI. An ongoing clinical trial (NCT02920788) is further evaluating GOALS training, including underlying neural mechanisms of change. The present study aimed to examine training-induced neuroplasticity by resting-state functional connectivity (rsFC) changes in GOALS versus active control. Veterans with a history of mTBI ≥6 months post-injury (*N* = 33) were randomly assigned to GOALS (*n* = 19) or an intensity-matched active control group (Brain Health Education [BHE] training; *n* = 14). GOALS consists of attention regulation and problem solving applied to individually defined, relevant goals through a combination of group, individual, and home practice sessions. Participants underwent multi-band resting-state functional magnetic resonance imaging at baseline and post-intervention. Exploratory 2 × 2 mixed analyses of variance identified pre-to-post changes in seed-based connectivity for GOALS versus BHE in five significant clusters. GOALS versus BHE demonstrated a significant increase in right lateral pre-frontal cortex connectivity with the right frontal pole and right middle temporal gyrus, as well as increased posterior cingulate connectivity with the pre-central gyrus. Rostral pre-frontal cortex connectivity with the right precuneus and the right frontal pole decreased in GOALS versus BHE. These GOALS-related changes in rsFC point to potential neural mechanisms underlying the intervention. This training-induced neuroplasticity may play a role in improved cognitive and emotional functioning post-GOALS.

## Introduction

Mild (mTBI) traumatic brain injury (TBI) is the most common type of TBI. International incidence of mTBI has been estimated at up to 600 per 100,000 or 42 million persons annually.^1^ Cognitive complaints are common post-mTBI and include difficulties with attention and various aspects of executive functioning such as working memory, cognitive flexibility, and problem solving.^2,3^ Although cognitive functioning is expected to quickly recover throughout the first few weeks post-injury, cognitive complaints often persist through 1 year post-injury and beyond^4,5^ and can impact well-being and interfere with working toward life goals. There is therefore a critical need for the prevention and treatment of cognitive difficulties in persons with a history of TBI, and interventions targeting such difficulties are emerging.

Goal-Oriented Attentional Self-Regulation (GOALS) training is one such treatment with demonstrated improvements in attention and executive functioning, as well as emotional functioning, among veterans with chronic TBI.^6–8^ An ongoing randomized controlled trial (NCT02920788) is further evaluating GOALS training among persons with a history of mTBI, including underlying neural mechanisms of change. To that end, the present study aims to examine treatment-related resting-state functional connectivity (rsFC) changes in persons with mTBI who underwent GOALS versus an active control group.

Resting-state functional magnetic resonance imaging (rsfMRI) measures spontaneous fluctuations in brain activity that occur synchronously across spatially distant regions.^9^ Functional connectivity analysis of rsfMRI data is useful for examining neural network changes in TBI given that multiple resting-state networks have shown disruptions in TBI, including the default mode, frontoparietal, salience, and dorsal attention networks. Extant work investigating TBI-related neural changes has much heterogeneity with respect to samples (e.g., TBI severity, time post-injury) and methods (e.g., image acquisition, mode of analysis), but findings point to both hypo- and hyperconnectivity within and between networks when compared to healthy controls.^10–16^

It has been suggested that a state of hyperconnectivity post-TBI may reflect brain network reorganization and help re-establish neural network communication in the short term, but may have negative consequences in the long term,^17^ for example by increasing risk for Alzheimer's disease.^18,19^ Neural changes related to mTBI have been shown to persist up to 10 years post-injury,^20^ yet importantly, continued neuroplasticity in the chronic phase of mTBI has also been documented.^21^ Further, increased integration of the resting-state functional connectome has been linked to improved cognitive recovery, especially attention and executive function, from the subacute to chronic stages of mTBI.^22^ Taken together, impaired neural network coordination may underlie difficulties in complex cognition. It is therefore important to expedite recovery and prevent long-term consequences of disrupted neural networks in the chronic phases of mTBI.

To address difficulties in complex cognition and improve functional outcomes, it has been suggested that cognitive rehabilitation in TBI should focus on attention and executive functioning training. Many cognitive rehabilitation interventions developed specifically for persons with TBI have focused on these domains,^23–25^ including GOALS. The core components of GOALS are 1) attentional self-regulation, a psychological process that incorporates mindfulness, redirection of attention, and filtering distractions, and 2) practicing these skills daily, in the context of personal goals.^6^ As such, it targets top-down cognitive pre-frontal cortex-mediated processes^26^ and has demonstrated cognitive, emotional, and functional improvements in veterans with chronic TBI^6,7^ as well as in veterans with mTBI and post-traumatic stress disorder (PTSD).^27,28^

Given documented cognitive and functional improvements after cognitive rehabilitation interventions, the underlying neural mechanisms (i.e., training-induced neuroplasticity) are now becoming a focus in research. Studies examining brain mechanisms of cognitive rehabilitation interventions have begun to document functional, structural, and neurophysiological changes after cognitive rehabilitation for persons with TBI across injury severity.^29–35^ Our previous work has documented evidence for training-induced neuroplasticity post-GOALS using task-based functional magnetic resonance imaging (fMRI).^33,36^ rsfMRI may be particularly useful for examining training-induced neuroplasticity after cognitive rehabilitation for TBI given its utility in examining neural network dysfunction in TBI, as well as evidence for changes in rsFC after cognitive training in healthy persons^37–39^ and in clinical non-TBI populations.^40,41^

A handful of studies have examined resting-state changes after cognitive rehabilitation in the chronic phases of TBI. A study by Han and colleagues found increased rsFC between areas in the default mode, somatomotor, and visual networks among a sample of participants with chronic TBI of mixed severity who underwent “Strategic Memory Advanced Reasoning Training” versus an active control group. Other smaller studies examining rsFC changes in adults with chronic TBI after cognitive rehabilitation training highlight similar preliminary insights that point to training-induced changes in rsFC within and between the default mode and task-positive networks.^42,43^ However, these studies are limited by very small sample sizes of ≤11 participants per group.

The present study examines training-induced neuroplasticity by rsFC in a randomized controlled study of GOALS training for veterans with a history of mTBI. To our knowledge, this is the first randomized controlled trial to examine cognitive rehabilitation-related rsFC changes in a sample consisting exclusively of mTBI patients in the chronic phase of recovery. Given the novelty of this research, we used an exploratory approach to identify pre-to-post changes between key resting-state network hubs and the rest of the brain. Seeds were selected from the default mode, frontoparietal, salience, and dorsal attention networks because these networks were previously shown to be disrupted in TBI and are thought to be targeted by GOALS training. Examining rsFC related to GOALS training may provide insight into the mechanisms underlying improvements in executive function among persons with mTBI. Given our exploratory analytical approach, we hypothesized broadly that changes in rsFC would distinguish persons with mTBI who underwent GOALS training versus an active control intervention.

## Methods

### Participants and design

This study was approved by institutional review boards at the University of California San Francisco and San Francisco VA Medical Center. All participants provided informed consent before any study procedures.

Participants were recruited from the San Francisco VA TBI Clinic, local VA community clinics, and veteran groups using institutional review board–approved information sheets and flyers. Inclusion criteria included: age ≥18; history of mTBI (>6 months post-injury, sustained either in combat or as a civilian); stable psychoactive medication regimen; report of moderate-to-severe residual cognitive difficulties (by Neurobehavioral Symptom Inventory) that interfere with daily function; and interest/availability to participate in cognitive training. History of mTBI was confirmed through Department of Defense/VA medical records and/or in-person Ohio State University TBI Instrument. Exclusion criteria included: history of moderate or severe TBI; unstable medical, neurological, or psychiatric conditions, including psychosis, severe PTSD, severe anxiety, or depression precluding participation in research activities such as assessment and/or training; contraindications to magnetic resonance imaging (MRI); illicit drug or alcohol use problems; or poor English comprehension.

**FIG. 1.**
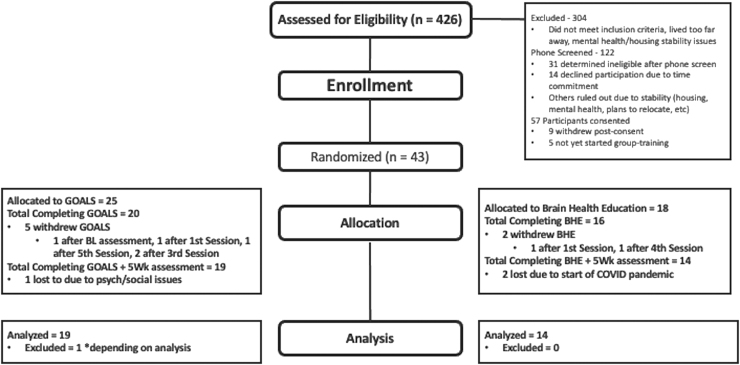
CONSORT flow diagram.

Figure 1 (CONSORT diagram) provides recruitment details. To summarize, 426 persons were assessed for eligibility and 57 consented to the study. Of those who consented, 9 withdrew post-consent but before randomization, and 5 are still active in the study but have not yet been randomized. Eight participants withdrew from the study because of scheduling/travel difficulties and/or family circumstances after being randomized. Two additional participants completed group training but were unable to complete post-training evaluations because of the COVID-19 shelter-in-place order and are therefore not included in analyses.

Thirty-three veterans completed study procedures and were included in the present analyses (24% female; mean age = 44.6 years [standard deviation {SD} = 14.2]; mean years education = 15.2 [SD = 2.35]). Eras of military service included Vietnam, Gulf War, and Operation Enduring Freedom/Operation Iraqi Freedom. TBIs were sustained from mixed causes, including blunt force injuries, motor vehicle accidents, as well as blast waves. History of more than one mTBI (e.g., military combat training, martial arts training, and high school football) was reported by 36% of participants. All participants were independent in basic activities of daily living but reported mild-to-moderate difficulties on tasks involving organization, problem solving, multi-tasking, and distractibility. Most participants were not working or going to school; 5 participants were gainfully employed and 10 were students.

After baseline evaluation, participants were placed in small groups, consisting of 2–3 participants of similar age, and the entire group was then randomized to receive either GOALS (*n* = 19) or Brain Health Education (BHE; *n* = 14). See Table 1 for a comparison of participants who participated in GOALS versus BHE. There were no differences in demographic or clinical characteristics at baseline.

**Table 1.** Demographic and Clinical Characteristics at Baseline

Variable	GOALS (*n* = 19)		BHE (*n* = 14)	Statistical comparison
Age, mean (SD)	41.1 (12.9)		49.5 (15.4)	0.12
Years of education, mean (SD)	15.1 (1.9)		15.5 (2.9)	0.67
Months since last injury, mean (SD)	118.0 (98)		176.3 (191)	0.31
Sex (% female)	21		29	0.42
Race (% non-White)	16		7	0.42
Hispanic ethnicity (%)	15		14	0.79
Overall Attention/EF z-score, mean (SD)	–0.05 (0.62)		–0.03 (0.66)	0.94
Mayo-Portland Total, mean (SD)	46.9 (14.4)		50.5 (6.7)	0.46
BDI-II Total, mean (SD)	18.8 (8.6)		18.8 (11.8)	1.00
PCL-M Total, mean (SD)	51.4 (11.7)		46.3 (17.5)	0.40

Overall Attention/EF represents the average z-score based on performance on multiple neuropsychological measures (Letter Number Sequencing; Auditory Consonant Trigrams 9, 18, and 36 sec; Digit Vigilance Test–Time and Errors; Design Fluency Switching; Verbal Fluency Switching; Trails B; Color-Word Interference Inhibition/Switching–Time and Errors; Color-Word Interference Inhibition–Time and Errors).

Missing data: years of education, *n* = 1 (GOALS); Attention/EF, *n* = 1 (GOALS); BDI-II and PCM-M, *n* = 7 (GOALS), *n* = 2 (BHE); statistical comparison column provides *p* values for statistical GOALS versus BHE group comparison (chi-square test for sex, race, and ethnicity and two-sample *t*-tests for all other variables).

SD, standard deviation; EF, executive functioning; PCL-M, Post-Traumatic Stress Disorder Checklist-Military Version; BDI-II, Beck Depression Inventory-II; GOALS, Goal-Oriented Attentional Self-Regulation; BHE, Brain Health Education.

Pre- and post-training, participants were evaluated with a multi-level battery consisting of neuropsychological and complex functional performance assessment and self-report measures of daily and emotional functioning. Assessments were administered by the same evaluator at both time points, and every attempt was made to administer them at the same time of the day. Evaluators were blinded to participants' treatment conditions, and evaluators and therapists were separate persons.

### Interventions

GOALS and BHE were matched closely for time with therapists and training intensity. Both were administered across ten 2-h group sessions, three 1-h individual sessions, and 20 hours of home practice across 5 weeks. Interventions were conducted in a small group format with 2–3 participants and two therapists per group. Intervention manuals were written for instructors and participants.^44^

#### Goal-Oriented Attentional Self-Regulation training

GOALS consists of attention regulation and problem solving applied to individually defined, relevant goals. For a detailed review of GOALS training, please see Novakovic-Agopian and colleagues.^6^ To summarize, the GOALS intervention emphasizes regulation of distractibility that is addressed by applied mindfulness-based attention regulation to redirect cognitive processes toward task-relevant activities even when distracted. Participants learn to use a metacognitive strategy (“Stop-Relax-Refocus”) to stop activity when distracted, anxious, and/or overwhelmed; relax; and then refocus attention on the current primary goal. They are taught to apply these skills actively to a range of situations, from simple information-processing tasks to challenging low-structure situations occurring in their own lives. Homework includes practice in maintaining goal direction during challenging real-life situations identified by participants.

#### Brain Health Education training

This is an active comparison matched with GOALS for time with therapists, homework load, and group and individual session participation hours. BHE training was designed to be engaging and provide information about brain functioning and brain health. Although session materials include information about effects of stress, sleep, and diet, they are educational in nature, emphasizing knowledge and not skills. Group leaders did not assist participants with making connections between the material presented and possible positive effects on their own daily functioning, or how to integrate into their daily lives. Further, the presumed active ingredients of GOALS training, which include applied problem solving and attention regulation, are not part of the BHE intervention.

### Neuropsychological and self-report measures

Participants were evaluated with a multi-level battery consisting of neuropsychological and self-report measures of daily and emotional functioning before and after GOALS or BHE. Similar to our previous studies,^6,7^ the current study used a neuropsychological battery specifically designed to assess performance in cognitive domains of complex attention and executive function that are commonly affected by TBI and targeted by GOALS training. The primary neuropsychological outcome, the Overall Attention/Executive Function composite, represents the average z-score based on performance on relevant neuropsychological domains and corresponding measures: Working Memory: Letter Number Sequencing; Auditory Consonant Trigrams^45^; Sustained Attention: Digit Vigilance Test–Time and Errors^46^; Mental Flexibility: Delis-Kaplan Executive Function System (D-KEFS) Design Fluency Switching; Verbal Fluency Switching; Trails B; D-KEFS Color-Word Interference Inhibition/Switching–Time and Errors; and D-KEFS Inhibition: Color-Word Interference Inhibition–Time and Errors.^47,48^ Participants also completed self-report measures of daily and emotional functioning, including the Mayo-Portland Adaptability Inventory,^49^ a measure of common sequelae of TBI including impact on activities of daily living, emotional adjustment, and community integration, the Beck Depression Inventory-II,^50^ and the PTSD Checklist, Military Version.^51^ Baseline levels of selected indices of these measures are provided in Table 1. Outcomes of these and other measures will be reported in a parallel article.

### Neuroimaging acquisition

Participants underwent MRI acquisition at baseline and again post-intervention (∼6 weeks later). All subjects were scanned on a 3.0 Tesla Siemens Skyra scanner (Siemens Medical Solutions USA, Inc., Malver, PA) at the SF VA Medical Center using a 32-channel head coil. rsfMRI data were acquired using an echo-planar imaging sequence with the following parameters: repetition time (TR) = 820 ms; echo time (TE) = 35 ms; flip angle = 52 degrees; number of slices = 72; slice thickness = 2 mm; spatial resolution = 2 mm^3^. Two runs of 366 brain volumes each were acquired for a total acquisition time of 10 min. Participants were instructed to open their eyes and remain awake during the rsfMRI scan sequences. Anatomical three-dimensional magnetization-prepared rapid acquisition with gradient echo scans were acquired using T1-weighting and the following parameters: TR = 2400 ms; TE = 2.24 ms; flip angle = 8 degrees; number of slices = 208; slice thickness = 0.8 mm; spatial resolution = 0.8 mm.^3^

### Functional magnetic resonance imaging data pre-processing

The CONN toolbox (version 17.f)^52^ was used for rsfMRI data pre-processing and run using MATLAB (version 2014a; The MathWorks, Inc., Natick, MA) and SPM12. All rsfMRI data were realigned and normalized to the Montreal Neurological Institute (MNI) template in SPM, slice-time corrected, and spatially smoothed with a 3-mm (full width at half maximum) Gaussian filter kernel. Normalization and coregistration were manually reviewed for quality control. Functional outlier detection was performed utilizing the ARtifact detection Tools–based identification of outlier scans using an intermediate setting for scrubbing. Additionally, CONN utilizes aCompCor to denoise the following confounds from the time-series data: 1) white matter and CSF signals; 2) motion parameters during the realignment process; and 3) volumes flagged for excessive motion from the scrubbing process. Each T1 anatomical image was also normalized to MNI space, coregistered, and automatically segmented into cerebrospinal fluid (CSF), gray matter, and white matter parcellations using SPM to utilize in the aCompCor denoising process.^53^ The data were finally bandpass filtered at 0.008–0.090 Hz to discern the rsfMRI data from low-frequency CSF, white matter, and motion signals.

### Resting-state functional magnetic resonance imaging data analysis

Broadly, analyses evaluated resting-state neural changes (pre- to post-treatment) for GOALS versus BHE. To that end, the CONN Toolbox was used to perform a seed-to-voxel functional connectivity analysis.

Subject-specific correlation matrices were computed using a Pearson correlation analysis to correlate the average blood oxygen level dependent time series of each seed region to the time series of each voxel of the brain. Coefficient maps were then Fisher-transformed into z-scores. We selected seed regions from CONN's default seeds that overall consist of 132 anatomical regions from the Harvard-Oxford Atlas and 32 seed regions from the eight recognized rsfMRI networks.^52^ Twenty seeds were selected based on anatomical regions and resting-state networks that were previously shown to be impacted by TBI, including regions in the frontoparietal (lateral pre-frontal cortex, posterior parietal cortex), salience (anterior cingulate cortex, anterior insula, rostral pre-frontal cortex, and supramarginal gyrus), dorsal attention (bilateral frontal eye field, and intraparietal sulcus), and default mode (medial pre-frontal cortex, precuneus cortex, and lateral parietal cortex) networks, as well as an additional relevant anatomical region that was not part of the Harvard-Oxford-Atlas–defined resting-state networks (orbital frontal cortex).

### Statistical analysis

Second-level 2 × 2 mixed analysis of variance (ANOVA) interaction analyses were performed to test Group × Time interactions in separate analyses for each seed. We investigated clusters that emerged at a two-sided threshold (voxel level: *p* < 0.005; cluster level: *p* < 0.05, false discovery rate [FDR] corrected). Effects of significant clusters were further examined by extracting each participant's averaged cluster values for *post hoc* analyses in SPSS software (SPSS, Inc., Chicago, IL). Paired-sample *t*-tests examined change over time within each group, two-sample *t*-tests evaluated group differences at each time point, and FDR correction was used to correct *post hoc* analyses for multiple comparisons within each cluster.

## Results

### Neuropsychological measures

A significant Group (GOALS vs. BHE) by Time (pre- vs. post-training) effect was identified for the primary neurocognitive outcome measure Overall Attention/Executive Function composite (*p* < 0.01), such that participants who received GOALS demonstrated more improvement after treatment compared to those persons who participated in the BHE intervention. Additional outcomes of behavioral measures are reported in a parallel article.

### Neuroimaging

**FIG. 2.**
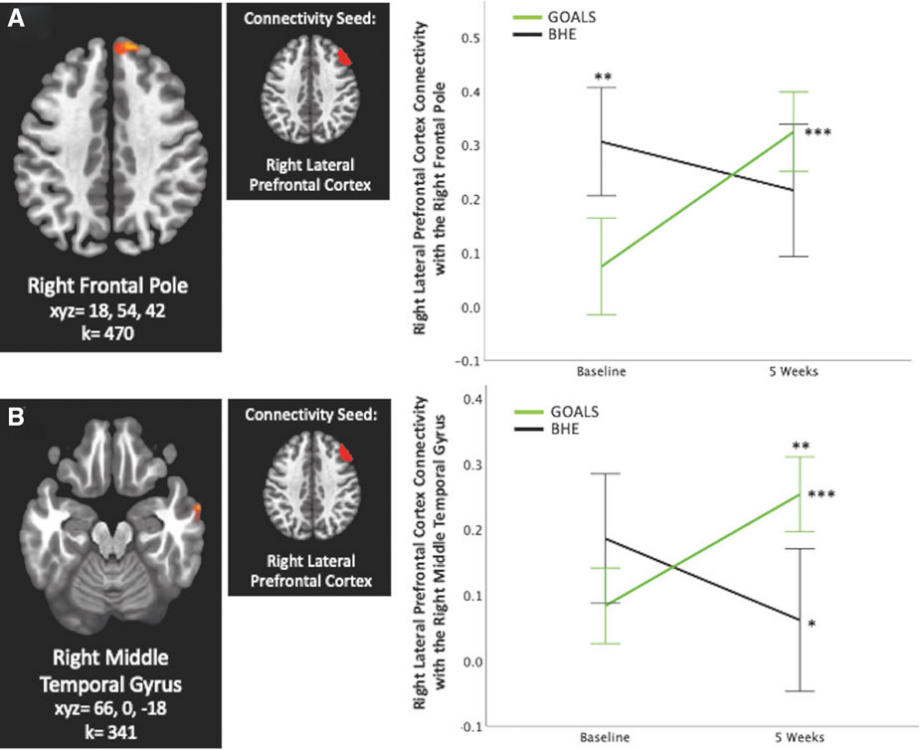
Lateral pre-frontal cortex connectivity. Group × Time interaction predicts right lateral pre-frontal cortex connectivity with the (**A**) right frontal pole and (**B**) right middle temporal gyrus. *p < 0.05, **p < 0.01, ***p < 0.001, corrected. Error bars represent 95% confidence interval. For this and all figures, GOALS = treatment group that underwent Goal-Oriented Attentional Self-Regulation, BHE = active control group that participated in an intensity-matched brain health education intervention. Brain images represent axial sections (left = left).

**FIG. 3.**
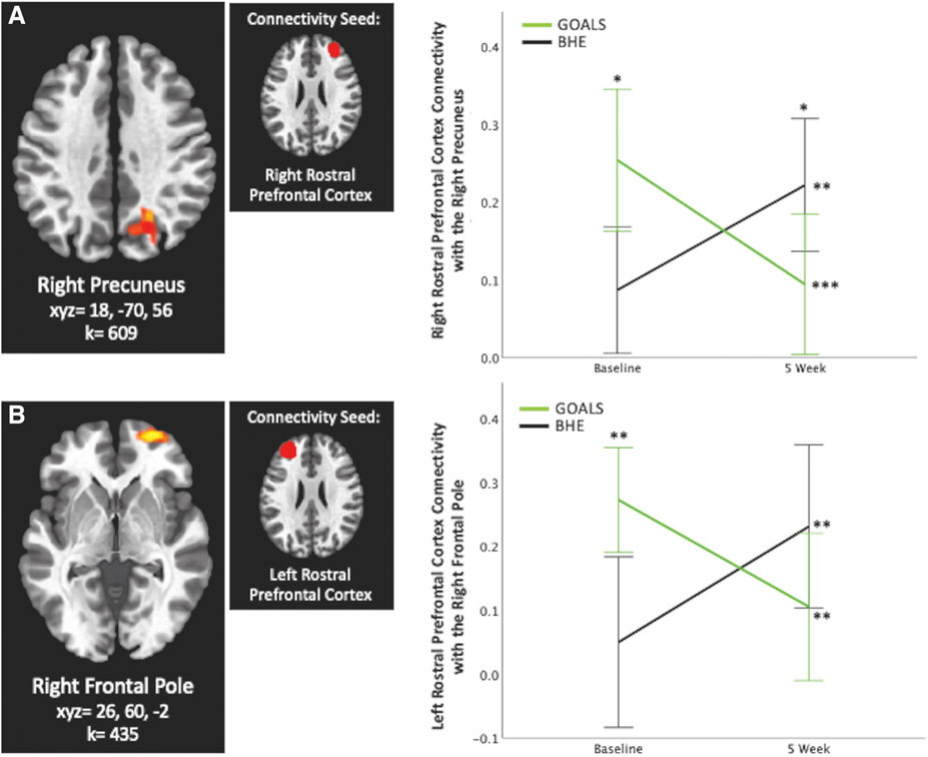
Rostral pre-frontal cortex connectivity. **(A**) Group × Time interaction predicts right rostral pre-frontal cortex connectivity with the right precuneus and (**B**) left rostral pre-frontal cortex connectivity with the right frontal pole. *p < 0.05, **p < 0.01, ***p < 0.001, corrected. Error bars represent 95% confidence interval.

**FIG. 4.**
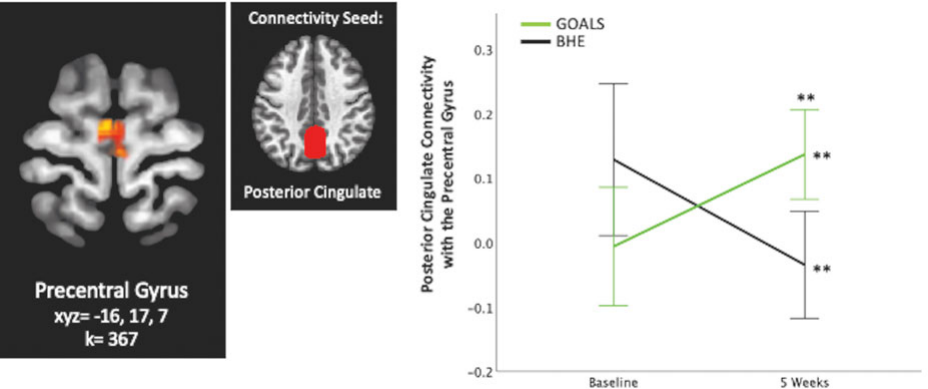
Posterior cingulate connectivity. Group × Time interaction predicts posterior cingulate connectivity with the pre-central gyrus. **p < 0.01, corrected. Error bars represent 95% confidence interval.

Overall, 5 significant clusters representing a significant Group × Time interaction effect emerged in our seed-based whole-brain functional connectivity analyses, with seeds in the left lateral pre-frontal cortex, left and right rostral pre-frontal cortex, and posterior cingulate. No other seeds yielded significant findings. Results are listed in Table 2, displayed in Figures 2–4, and described below. Overall, GOALS and BHE groups demonstrated differential changes i2n connectivity over time.

**Table 2.** Significant Clusters of Interest Resulting from Seed to Voxel Analyses

Seed region	k	F_1.31_	x	y	z	BA	Connectivity cluster region
Right lateral pre-frontal cortex							
	470	25.0	18	54	42	8	Right frontal pole
	341	34.9	66	0	–18	21	Right middle temporal Gyrus
Right rostral pre-frontal cortex							
	609	33.4	18	–70	56	7,31	Right precuneus
Left rostral pre-frontal cortex							
	435	31.4	26	60	–2	10	Right frontal pole
Posterior cingulate cortex							
	367	21.1	–4	–12	70	6	Bilateral pre-central gyrus

k represents the number of voxels in a given cluster; *F* statistic represents *post hoc* Time × Group effect; xyz values are provided in MNI coordinates and represent voxel of peak activation.

BA, Brodmann areas; MNI, Montreal Neurological Institute.

### Lateral pre-frontal cortex connectivity

Group × Time significantly predicted right lateral pre-frontal connectivity with the right frontal pole (k = 470, *F*_1,31_ = 25.0, xyz = 18, 54, 42; Fig. 2A) and with the right middle temporal gyrus (k = 341, *F*_1,31_ = 34.9, xyz = 66, 0, −18; Fig. 2B). FDR-corrected *post hoc* analyses indicated that connectivity between these areas significantly increased in GOALS over time (*p* < 0.001). BHE demonstrated no change in right lateral pre-frontal connectivity over time with the right frontal pole cluster and a decrease over time with the right middle temporal gyrus cluster (*p* < 0.05).

### Rostral pre-frontal cortex connectivity

Group × Time significantly predicted right rostral pre-frontal connectivity with the right precuneus (k = 609, *F*_1,31_ = 33.4, xyz = 18, −70, 56; Fig. 3A) and left rostral pre-frontal connectivity with the right frontal pole (k = 435, *F*_1,31_ = 31.4, xyz = 26, 60, −2; Fig. 3B). FDR-corrected *post hoc* analyses indicated that connectivity between these areas significantly decreased in GOALS over time (right precuneus: *p* < 0.001; right frontal pole: *p* < 0.01), whereas BHE demonstrated an increase in connectivity between these areas over time (*p* < 0.01).

### Posterior cingulate connectivity

Group × Time significantly predicted posterior cingulate connectivity with the right pre-central gyrus (k = 367, *F*_1,31_ = 21.1, xyz = 4, −12, 70; Fig. 4). FDR-corrected *post hoc* analyses indicated that connectivity between these areas significantly increased in GOALS over time (*p* < 0.01). BHE demonstrated a decrease in connectivity between these areas over time (*p* < 0.01).

## Discussion

GOALS training, a cognitive rehabilitation intervention that incorporates mindfulness and redirection of attention in the context of personal goals, has shown to improve executive functioning in previous TBI samples^6,7^ as well as in the current sample. The present study aimed to examine training-induced neuroplasticity in a randomized controlled study of GOALS for mTBI. To this end, we examined pre- to post-treatment rsFC changes in persons who underwent GOALS versus BHE, an active control group. Our exploratory analyses revealed significant rsFC changes between neural network regions related to attention and executive functioning. Connectivity increased or decreased among GOALS versus BHE depending on the seed region. There was an increase in connectivity from baseline to post-intervention between hubs of the frontoparietal and default mode networks and frontal and temporal regions, and a decrease in connectivity between salience network hubs and parietal and frontal regions. Although it will be important to conduct formal mediation analyses in larger samples to test this hypothesis, these findings suggest that improved functioning among patients with mTBI who underwent GOALS training may work through synchronization of communication between task-negative and -positive networks underlying attention and executive processes.

We demonstrated training-induced neuroplasticity through changes in rsFC using key hubs of the frontoparietal, salience, and default mode networks as connectivity seeds. Increased rsFC emerged among the GOALS groups versus BHE between the right lateral pre-frontal cortex, a key area of the frontoparietal network, and the right frontal pole and right middle temporal gyrus. These results align with findings by Han and colleagues,^31^ who examined rsFC among persons with chronic TBI who underwent a strategy-based reasoning training that included training in selective attention and thinking strategies versus an active control group. Despite a different analytical approach, their analyses revealed several similar patterns when comparing the cognitive rehabilitation group to active control, specifically increased rsFC between the right dorsal/lateral pre-frontal cortex and right anterior pre-frontal cortex, as well as between the right dorsal pre-frontal cortex and right middle temporal areas. The frontal pole and temporal areas that emerged in our analyses, as well as the areas described by Han and colleagues,^31^ overlap with regions encompassed in the default mode network as defined by the Yeo resting state network atlas,^54^ suggesting increased connectivity between the frontoparietal and default mode networks.

Altogether, this could reflect increased efficiency in the ability to switch between automatic processes of the default mode network to controlled effortful processes thought to be mediated by the frontoparietal network. Notably, these frontoparietal network findings are limited to the right hemisphere, which agrees with previous work that demonstrated increased connectivity limited to the right frontoparietal network.^12^

We found decreased rsFC in the GOALS group relative to the active control group in our analyses using the rostral pre-frontal cortex, a region within the salience network, as a seed. Specifically, rsFC connectivity decreased between the right rostral pre-frontal cortex and the right precuneus, as well as between the left rostral pre-frontal cortex and the right frontal pole. An overlap of the specific anatomical regions that encompass the resulting connectivity clusters with the Yeo resting-state network atlas^54^ suggests that the specific right precuneus and right frontal pole regions that emerged here overlap with regions in the default mode/dorsal attention networks and the frontoparietal network, respectively. The salience network has been shown to support shifting between internally and externally directed states and may therefore be important for regulating network functioning to support efficient cognition. Decreased connectivity between salience and other networks may enable increased efficiency of network-specific tasks and support improvements in complex cognitive efforts requiring attention and executive functions.

Our findings further point to increased rsFC between the posterior cingulate, the dorsal hub of the default mode network, and the pre-motor cortex, an area typically associated with task-positive networks. This accords with other studies of rsFC changes after cognitive rehabilitation in chronic TBI. Both Gimble and colleagues and Lindsey and colleagues found increased connectivity between task-negative and -positive network hubs among persons with TBI who underwent cognitive rehabilitation versus waitlist controls. Increased connectivity between these areas may indicate improved coordination and may facilitate attentional switching between automatic and effortful processes.

This study adds to our previous work and begins to establish an understanding of the neural mechanisms that may underlie GOALS training.^33,36,55^ For example, Chen and colleagues^33^ demonstrated enhanced modulatory control over visual processing in the context of task-based fMRI post-GOALS in a sample of patients with acquired brain injuries (75% TBI). They suggested that these findings may represent a rebalancing of pre-frontal functioning that may underlie improvements in attention and executive control. The training-induced increases and decreases in rsFC over time in the present study may likewise point to a rebalancing of neural networks.

It is important to note that in addition to significant changes in rsFC among the GOALS group, many of our findings also demonstrated significant rsFC changes in the BHE active control group, but in the opposite direction. The BHE group underwent the intensity-matched active control condition consisting of classes on brain functioning and brain health without providing a link between the material and application in daily life. Intensity-matched home practice in the active control group consisted of watching or reading informational material, whereas one of the key aspects of GOALS is to practice the skills in daily life. Therefore, both groups, GOALS and BHE, experienced a change to their daily routine by incorporating a rigorous and time-consuming program into their lives for 5 weeks; however, the essence of how their routines changed differed notably. Significant changes in cognitive performance were only observed among the GOALS group, whose overall attention/executive functioning performance improved from baseline to post-intervention. Our analyses may have therefore captured the network changes that differentiate incorporating active acquisition and practice of skills applied to personal goals versus merely acquiring knowledge in the context of participating in a time-intensive research study.

The present findings are limited by several factors. Given our small sample size (*N* = 33), we were underpowered to examine whether connectivity changes relate to changes in cognition and symptoms. We were also only powered to detect large treatment effects, which prevented us from detecting potentially clinically meaningful medium or smaller treatment effects. Finally, in some clusters, we observed significant baseline connectivity differences between groups, which may emerge by chance in small samples such as ours. Replication of our findings in larger multi-center studies that also permit generalization to diverse populations is therefore needed. Such studies will also enable meaningful evaluation of the relation between neural connectivity and changes in cognition and symptoms. Until then, it is important to evaluate the interpretations offered here with caution.

## Conclusion

The current study provides evidence of training-induced neuroplasticity in veterans with mTBI after GOALS training. It is possible that the observed changes in rsFC underlie improvements in emotional and cognitive functioning, although future studies must examine this hypothesis in larger samples powered to examine these relations.

## Transparency, Rigor, and Reproducibility Statement

The study was pre-registered at clinicaltrials.gov (NCT02920788). The analysis plan was not formally pre-registered. A sample size of 62 subjects was planned, and the sample size for the present study is 33 subjects: 19 in the treatment group and 14 in the active control group. Enrollment fell short of the target because of the COVID-19 pandemic. Four hundred twenty-six potential participants were screened; imaging data were obtained from 43 and successfully analyzed in 33 who completed treatment, provided pre- and post resting-state data, and met data quality-assurance criteria. Imaging acquisition, imaging quality-control decisions, and analyses were performed by team members blinded to treatment allocation of participants. All equipment and software used to perform imaging and pre-processing are widely available from commercial sources. The key inclusion criteria and outcome evaluations are established standards. Non-independence of measurements has been addressed using mixed ANOVA models. Correction for multiple comparisons was performed using false discovery rate.

At this time, no replication or external validation studies have been performed or are planned/ongoing to our knowledge, but our team is currently in the early stages of planning a replication and validation study. Deidentified data from this study are not available in a public archive. Deidentified data from this study will be made available (as allowable according to institutional IRB standards) by e-mailing the corresponding author. Analytical code used to conduct the analyses presented in this study are available through the CONN toolbox, which was used for the present analyses. The authors agree to provide the full content of the manuscript on request by contacting the corresponding author.

## Abbreviations Used

ANOVA: analysis of variance
BHE: Brain Health Education
CSF: cerebrospinal fluid
D-KEFS: Delis-Kaplan Executive Function System
FDR: false discovery rate
fMRI: functional magnetic resonance imaging
GOALS: Goal-Oriented Attentional Self-Regulation
MNI: Montreal Neurological Institute
MRI: magnetic resonance imaging
mTBI: mild TBI
PTSD: post-traumatic stress disorder
rsFC: resting-state functional connectivity
rsfMRI: resting-state functional magnetic resonance imaging
SD: standard deviation
TBI: traumatic brain injury
TE: echo time
TR: repetition time
